# Revalidating the Taiwanese Self-Regulation Questionnaire (New TSSRQ) and Exploring Its Relationship With College Students’ Psychological Well-Being

**DOI:** 10.3389/fpsyg.2020.01192

**Published:** 2020-06-19

**Authors:** Yang-Hsueh Chen, Yu-Ju Lin

**Affiliations:** ^1^Institute of Teacher Education, National Chengchi University, Taipei, Taiwan; ^2^Teaching and Learning Technologies, Purdue University, West Lafayette, IN, United States

**Keywords:** self-regulation, factor analysis, measurement, college students, psychological well-being

## Abstract

Self-regulation (SR) is a vital trait whereby people adapt to the environments and achieve goals, yet measurements of general SR remain scant in Asian countries. Due to insufficient items in several dimensions, in this study we revised and revalidated our previous work of the Short Self-Regulation Questionnaire for Taiwanese college students (TSSRQ) by incorporating student perspectives and aspects of affective/motivation regulation. Through exploratory and confirmatory factor analyses, we validated the “New TSSRQ” which contained 39 items in seven factors, including *Proactiveness* (PA), *Self-Management* (SM), *Goal Setting* (GS), *Mindfulness* (MF), *Goal Attainment* (GA), *Adjustment* (AD), and *Motivation* (MO). Subsequently, we explored the correlation between New TSSRQ dimensions and those of the Scale of Psychological Well-Being (SPWB) as a source of validity evidence. Findings indicated that SR and PWB are highly correlated, especially for *Mindfulness* and *Proactiveness* dimensions. Implications of this study were discussed along with practical suggestions to leverage college students’ mindfulness, proactiveness, and self-regulation in general.

## Introduction

Self-regulation (SR) has been regarded as an important trait whereby people adapt their feelings, thoughts, actions, motivation, and so on in order to achieve their goals (Zimmerman, 1998; Schunk, 2008). If an individual can regulate his/her conduct effectively, s/he is more likely to maintain positive and healthy functioning as opposed to maladaptive behaviors (Pichardo et al., 2018). SR has been applied to a variety of issues and settings, such as addiction (Taipale, 2017), religion (Laurin and Kay, 2016), workplace motivation (Grandey, 2000), intimate relationships (Overall et al., 2006), crime (Tittle et al., 2003), sports (Nicholls, 2016), and learning (Boekaerts, 1997). When students apply SR at school, they use meta-cognitive strategies to plan, monitor, and modify their learning and cognition (Brown, 2011), including management skills for academic performance (Pintrich and DeGroot, 1990). Such a learning process is termed self-regulated learning (SRL).

In the SR/SRL literature, a strand of study examines the effects of SR on individuals’ psychological well-being (PWB) and related outcomes. For example, Mattern and Bauer (2014) found a positive relationship between job satisfaction and teachers’ SR. In another study, teachers’ SR was negatively related to their emotional exhaustion. On the other hand, Balkis and Duru (2016) found that college students’ lack of SRL skills was associated with procrastination and lower levels of academic satisfaction and affective well-being. More recently, Singh and Sharma (2018) found that young adults’ SR capacity positively correlated with their PWB, as measured by Carey et al.’ (2004) short version of the Self-Regulation Questionnaire (SSRQ) and Ryff and Keyes (1995) Scale of Psychological Well-Being (SPWB).

Over the years, many SR/SRL models have been proposed (see Panadero, 2017, for a review), wherein important elements have been identified (usually in phases). In the application of SR, three SR processes are addressed in social cognitive theory: *Self-judgment*, *Self-observation* (self-monitoring), and *Self-reaction* (Schunk, 2001, 2008). These three self-regulated processes align with Zimmerman’s (1998) three-phase SR model: *Forethought*, *Performance* or *Volition control*, and *Self-reflection*. Likewise, Pintrich’s (2000) proposed an SRL model that contains four stages: (1) *Forethought, Planning and activation*; (2) *Monitoring*; (3) *Control*; and (4) *Reaction and reflection*. In each stage, the individuals regulate their cognition, motivation, and behaviors; meanwhile, they adjust their actions and reactions according to contextual specifications. Behind SR, Pintrich’s (2004) identified four basic assumptions, including: (1) active/constructive nature of human beings, (2) individuals’ potential for self-monitoring and control, (3) goal/criterion-based evaluation for behavioral adjustments, and (4) SR as a mediator between personal and contextual characteristics and actual performance.

### Measurement of Self-Regulation

Regarding measurement, several quantitative instruments have been developed based on different SR/SRL models, such as *Motivated Strategies for Learning Questionnaire* (MSLQ; Pintrich et al., 1991), *Self-Directed Learning Readiness Scale* (SDLRS; Guglielmino, 1977), *Self-regulation Questionnaire* (SRQ; Brown et al., 1999), and the *Short Self-regulation Questionnaire* (SSRQ; Carey et al., 2004). The MSLQ (81 items) was created by Pintrich et al. (1991) to measure students’ motivation (expectancy, value, and task anxiety) and their cognitive, metacognitive, and resource management strategies (e.g., rehearsal, organization, and help seeking) as they study in a specific course. The SDLRS (58 items), on the one hand, was developed by Guglielmino (1977) to measure students’ attitudes, abilities, and characteristics that constitute their readiness to engage in self-directed learning. The SDLRS contains eight dimensions, such as *Openness to learning opportunities and Love of learning*. While both MSLQ and SDLRS are useful for measuring specific actions and strategies that students apply in a learning environment, they are less applicable to issues and contexts outside of learning.

Contrasting MSLQ and SDLRS, another strand of measurement assesses individuals’ SR in a general manner. As the first attempt to measure trait SR, Miller and Brown (1991) created the 63-item SRQ (e.g., Once I have a goal, I can usually plan how to reach it) based on their seven-stage theorizing. Subsequent studies (e.g., Gavora et al., 2015) have validated the SRQ, of which Carey et al. (2004)’s work was most cited. In that study, Carey et al. (2004) examined the psychometric properties of SRQ with a sample of 391 college students in the United States. Exploratory factor analysis (EFA) yielded only one factor with 31 items, which explained 43% of total variance. These 31 items were retained to create a short form Self-Regulation Questionnaire abbreviated as SSRQ.

The SSRQ has inspired follow-up revalidation studies conducted in different countries with different participant groups (see Chen and Lin, 2018, for a review), including Neal and Carey (2005), Potgieter and Botha (2009), Vosloo et al. (2013), and Garzón Umerenkova et al. (2017). Overall, these revalidation studies yielded different dimensions and numbers of factors, reflecting that the constitution of SR may vary by participant groups and culture (Vosloo et al., 2013; Garzón Umerenkova et al., 2017), and that validation studies are warranted to better capture SR of a group of people in a given setting (Chen and Lin, 2018). In view of the fact that validation studies of SSRQ remain scant in Asian countries, in our previous study (Chen and Lin, 2018) we revalidated the SSRQ with 1,998 college students across Taiwan (later, we named it as “TSSRQ”). Via EFA and confirmatory factor analysis (CFA), we retained 22 items in five factors, including *Goal Attainment* (GA, seven items), *Mindfulness* (MF, seven items), *Adjustment* (AD, three items), *Proactiveness* (PA, three items), and *Goal Setting* (GS, two items). These five factors explained 54% of the total variance.

### Gaps, Purposes, and Research Questions

Despite having revalidated a 5-factor TSSRQ in our previous study, two research gaps were identified. First of all, some dimensions only contained two to three items that undermined their representativeness (Beavers et al., 2013). Worse, the 22 items were somewhat dated, which could be traced back to Miller and Brown’s (1991) SRQ which is almost 30 years old. Secondly, the association between trait SR and PWB remains unclear among Taiwanese college students. While to date a number of SR–PWB correlation studies have been identified, none of them were assessed based on Taiwanese samples. Also notable is that existing studies merely report the correlations between SR and PWB total scores (e.g., Singh and Sharma, 2018), yet very few studies further examine the association between SR and PWB at dimension level. In view of the above research gaps, the purposes of this study are two-fold: first, to revalidate the TSSRQ (we term it as “New TSSRQ”) that expands SR items and perspectives; secondly, to explore the association between Taiwanese college students’ SR and PWB, which may also serve as a source of validity evidence of the New TSSRQ. Two research questions were proposed to guide this study:

RQ1: What are the dimensions of the New TSSRQ?
RQ2: What is the relationship between Taiwanese college students’ New TSSRQ scores and their psychological well-being, as a source of validity evidence?

## Materials And Methods

This study contains three stages. As will be detailed in later sections, in the ***First Stage: Item Creation*** we generate New TSSRQ items via literature review and collections of college students’ viewpoints about SR, especially for *Goal Setting*, *Proactiveness*, and *Adjustment* perspectives. Next, in the ***Second Stage: Dimension Validation*** we conducted a national survey across Taiwan to validate the items and dimensions. In the ***Third Stage: PWB Correlation*** we explored the correlation between Taiwanese college students’ SR and PWB in order to fill the aforementioned research gaps and to further examine the concurrent validity of the New TSSRQ. We expected that, through this three-stage study, the newly validated TSSRQ could better reflect the current context and culture among Taiwanese students toward SR, meanwhile, to help us explore its relationship with PWB and its future applications.

### Participants

The target participants were college students in Taiwan. In the ***First Stage: Item Creation*** 62 participants were recruited from one public (*N* = 26) and a private (*N* = 36) university in Northern Taiwan to provide open-ended opinions on SR. In the second stage national survey, the participants were recruited from the Northern, Middle, Southern, and Eastern parts of Taiwan as well as the outlying islands to ensure representativeness of data. Because college students’ study majors include nine main domains (see Table 1), we deemed it more practical to find the courses that include students from diverse academic backgrounds and study majors. Therefore, we contacted six general education or teacher education centers in selected universities and obtained their permission to administer the TSSRQ items. In turn, we obtained 969 valid participants across Taiwan for subsequent dimension validation analyses. As with the ***Third Stage: PWB Correlation***, we recruited another convenience sample of 532 (240 male, 281 female, and 1 unanswered) college students from both public (*N* = 317) and private (*N* = 215) universities. These students primarily study at the colleges in Northern Taiwan. The demographic profiles of participants in the second and third stages are presented in Table 1.

**TABLE 1:** Demographic profiles of the participants in the second and third stages of the national survey.

		**Second stage**		**Third stage**	
		***N***	**Percentage (%)**	***N***	**Percentage (%)**
Gender	1. Male	356	36.8	240	45.1
	2. Female	610	63.1	291	54.7
	Unanswered	3	0.1	1	0.2
	Total	969	100	532	100
Grade level	1. Freshman	300	31	60	11.3
	2. Sophomore	231	23.8	150	28.2
	3. Junior	218	22.5	136	25.6
	4. Senior and above	218	22.5	183	34.4
	Unanswered	2	0.2	3	0.6
	Total	969	100	532	100
Study major	1. Education	122	12.6	74	13.9
	2. Humanities and arts	234	24.1	155	29.1
	3. Social sciences, business, and law	206	21.3	53	10
	4. Science	69	7.1	31	5.8
	5. Engineering, manufacturing, and construction	81	8.4	138	25.9
	6. Agriculture	13	1.3	0	0
	7. Health and welfare	57	5.9	4	0.8
	8. Services	43	4.4	0	0
	9. Other/miscellaneous	136	14	74	13.9
	Unanswered	8	0.8	3	0.6
	Total	969	100	532	100

### Instrumentation

There are two instruments in this study, including (1) the *Short Self-Regulation Questionnaire for Taiwanese college students* (TSSRQ) to be expanded/revalidated in this study, and 2) the *Scale of Psychological Well-Being* (SPWB), which has often been cited in well-being studies. In this section, we present the “New-TSSRQ” item creation process, followed by more detailed descriptions of SPWB.

#### TSSRQ: Item Creation

Self-regulation can be defined as a vital trait whereby people adapt their feelings, behaviors, and thoughts or motivation into the environment to achieve goals (Zimmerman, 1998; Schunk, 2008). In the original TSSRQ (Chen and Lin, 2018), three dimensions (i.e., Goal Setting, Adjustment, and Proactiveness) only contained two or three items, which runs the risk of under-representativeness of latent factors (Beavers et al., 2013). Accordingly, a main task in this TSSRQ revision was to expand items in the GS (ability to plan and set clear goals), GA (action to track progress to achieve goals), and PA (actively seek possibilities to change something) dimensions. Taking a bottom-up point of view (McConaughy, 2001; Jain et al., 2013), or what Pintrich’s (2004) called “student approaches to learning, SAL,” we designed open-ended questions asking Taiwanese students’ perceptions of the following four questions:

1. **General trait self-regulation:** In your opinion, can you describe the general characteristics and behaviors of a self-regulated college student?
2. **Goal setting:** For a college student, what do you think a self-regulated college student will do when setting goals?
3. **Adjustment:** What will self-regulated college students do when they make a mistake or encounter challenges and difficulties?
4. **Proactiveness:** According to your observation, could you describe a proactive college student’s behavior?

Upon collecting college students’ (*N* = 62) responses, we organized them into subcategories and then elaborated representative/most frequent responses into scale items. For instance, under **Question 4**. **Proactiveness** we obtained subcategories of “*Expansions of Perspectives*,” “*Active Learning*,” and “*Other Characteristics*”; then, we converted representative responses into items such as “I actively develop multiple interests,” “I keep learning actively to improve myself,” and “I can do things well without others’ reminders,” respectively. We deem that the inclusion of student opinions of SR not only helped expand the existing item pool in GA, GS, and PA, but also more importantly, it enriched the content of items that better capture the perspectives and culture of Taiwanese college students.

Furthermore, the previous TSSRQ mainly focuses on cognitive but somewhat neglects affective aspects of SR. Therefore, in this revision we surveyed several SR/SRL frameworks in search of motivational/affective aspects of SR, such as Guglielmino’s (1977) *Self-directed Learning Readiness* and Pintrich’s’s (2004) framework for assessing motivation and SRL. We determined to consult Pintrich’s’s (2004) framework that elaborates on individuals’ selection and adaptation of strategies for managing motivation and affect, such as *positive self-talk*, *setting extrinsic rewards*, and *controlling emotions* (Ljubin-Golub et al., 2019). A couple of items were drafted, such as “When I am lacking confidence or motivation, I give myself pep talk to empower myself,” and “I would set rewards or punishments for myself to increase my motivation on completing task.” Together, we generated 50 new items out of open-ended questions and literature review, which comprised 14 Motivation (MO) items, 9 Goal Setting (GS) items, 8 Adjustment (AD) items, and 8 Proactiveness (PA) items. Other than these, we sorted 11 items that belonged to general characteristic of SR. The 50 new items were then combined with the original 22 TSSRQ items, making the total item pool 72 in number. Two undergraduate student assistants helped check the language and wording of these items. We also invited three scholars with the backgrounds in Educational Psychology and Cognitive Psychology to review items in order to ensure the logical flow and content validity. For the following dimension validation, please see the “Results” section.

#### Scale of Psychological Well-Being (SPWB)

As with Taiwanese college students’ PWB, we applied the 42-item SPWB (Ryff and Keyes, 1995) to measure the multiple facets of PWB, including (1) ***Autonomy (AT)***, which refers to the internal status of being independent and self-determined. A sample question is, “I am not afraid to voice my opinions, even when they are in opposition to the opinions of most people.” (2) ***Environmental Mastery (EM)*** means competence in managing the environment and making use of opportunities and resources to fit personal needs. A sample question is, “The demands of everyday life often get me down (reversely coded).” A person who scores higher on (3) ***Personal Growth (PG)*** sees him or herself as growing and expanding. A sample item is, “When I think about it, I haven’t really improved much as a person over the years (reversely coded).” (4) ***Positive Relations with Others (PR)*** means a person has established trust and satisfying relationships with others, as evidence by the sample question, “I enjoy personal and mutual conversations with family members or friends.” (5) ***Purpose in Life (PL)*** describes a person who has goals and objectives for living, and sample question is, “I enjoy making plans for the future and working to make them a reality.” Lastly, (6) ***Self-acceptance (SA)*** is a mental status that a person possesses a positive attitude toward the whole self, including strengths and weaknesses. A sample is, “When I compare myself to friends and acquaintances, it makes me feel good about who I am.” According to Seifert (2005), the Cronbach’s alphas for SPWB subscales were 0.86 for AT, 0.90 for EM, 0.87 for PG, 0.91 for PR, 0.90 for PL, and 0.93 for SA. In this study, the Cronbach’s alphas were 0.74 for AT, 0.73 for EM, 0.74 for PG, 0.78 for PR, 0.80 for PL, 0.80 for SA, and 0.93 for the total scale, indicating acceptable internal consistency of SPWB. To be consistent, all of the items in this study were rated on a 7-point Likert scale from (1) *very untrue* to (7) *very true*.

### Data Collection and Analysis

Regarding data collection, an ethics approval was not required as per institutional and national guidelines and regulations. In addition, it was entirely voluntary for students to participate in the anonymous survey, and consent was obtained upon the survey completion.

As mentioned earlier, this study contains three stages. In the ***First Stage: Item Creation***, the open-ended questions were delivered both online (via the mail list of a teacher education center at a public university) and through paper-based questionnaires (sent to an instructor at a private university). In the ***Second Stage: Dimension Validation*** and the ***Third Stage: PWB Correlation***, we used paper-based format only. Bundled survey questionnaires were mailed to the program administrators or directly to the instructors. Then, they brought the questionnaires to the class and explained the purpose of the study. Students who agreed to participate went on to complete the anonymous survey, while those who were unwilling to participate could leave it blank without any forms of penalty. After students completed the questionnaires, the administrators and instructors mailed them back to the researchers. Small gifts (a ball pen) were given to each participant in all three stages of data collection.

Regarding data analysis, in order to answer **Research Question 1**, “What are the dimensions of the New TSSRQ?”, we started with item analysis which included an examination of the mean, standard deviation, skewness, and kurtosis of each item. In addition, participants were sorted into “high” and “low” groups based on the 27 and 73 percentile ranks of their New TSSRQ total scores. Next, we randomly selected one third of the sample (*N* = 323) for EFA. Bartlett sphericity test and the KMO index were conducted with SPSS 22.0 to determine that the data was appropriate for factor analysis. During EFA, one was set as the threshold of eigenvalue to determine the number of factors/dimensions.

The remaining two-thirds of the sample (*N* = 646) was used in CFA to verify the dimensions generated by EFA. We utilized the *Mplus* program with the Maximum Likelihood estimation. Model fit, as suggested by Jackson et al. (2009), was measured by Chi-square fit index (χ^2^), comparative fit index (CFI), root mean square error of approximation (RMSEA), and the standardized root mean square residual (SRMR). CR and AVE analyses were applied to verify the convergent and discriminant validity of the dimensions. Lastly, the reliability test was conducted to calculate Cronbach’s alphas of the subscales and the total scale was calculated in order to determine the internal consistency of New TSSRQ.

In order to answer **Research Question 2**: “What is the relationship between Taiwanese college students’ self-regulation and psychological well-being, as a source of validity evidence?”, we adopted Pearson correlation analysis to explore the association between New TSSRQ and SPWB dimensions and total scores. Such an analysis not only helps us understand the direction and strength of the relationships between the two important constructs, but it is also helpful to assess the concurrent validity of the New TSSRQ. In addition, we conducted independent sample *t*-tests to explore gender difference in each TSSRQ and SPWB dimension.

## Results

### Validation of New TSSRQ Dimensions

In the ***Second Stage: Dimension Validation***, we proceeded with item analysis, EFA, CFA, and reliability test, as detailed below:

#### Item Analysis

Descriptive statistics result for the New TSSRQ (72 items) showed that the mean scores of each item lay between 4.71 and 5.70, and the standard deviations were mostly above 0.90 except Mindfulness (*SD* = 0.69) and Goal Setting (*SD* = 0.87). The maximum value of the item skewness was 1.26 in absolute value, indicating good dispersion of scores across all the items. In addition, participants were sorted into “high” and “low” groups. Independent sample *t*-test results showed that, for each of the 72 items, the high and low groups differed significantly at 0.001 level, indicating good item discrimination. We further calculated the correlation between the item and total score in the dimension (after being corrected to eliminate the effect of the item in the total score). Results showed that all the item-total correlations were above 0.30; therefore, all the items were retained.

#### Exploratory Factor Analysis (EFA)

Before conducting EFA, we completed the Bartlett sphericity test (χ^2^ = 16,922, *df* = 2556, *p* < 0.001) and calculated the KMO index (0.948) to determine that the data were appropriate for factor analysis. We conducted three rounds of EFA with the *Mplus* program (using Maximum Likelihood method with GEOMIN Oblique rotation) to delete items and determine factors. A total of 11 eigenvalues are over 1 and indicate a possible 11 factors. Through Parallel Analysis with SPSS 22.0, we determined to retain 11 factors. As Hair et al. (1995) pointed out that factor loading of 0.5 can be viewed as being significant in practice (in comparison with factor loading of 0.4 being considered important), we decided to adopt 0.5 as the threshold to keep or remove items. Therefore, in the second round, eight factors with 44 items were generated. We checked the interpretation of individual items within each factor. In the third round, we further removed one factor which simply included two items under *Mindfulness* and two items within *Goal Attainment*. Therefore, seven factors with a total of 39 items, named New TSSRQ, were attained (see Table 2 and Appendix) and achieved the explained 55.52% of the total variance. More details of the factors are described below:

**TABLE 2:** Factor structure and factor loadings of the new Taiwanese Short Self-regulation Questionnaire (new TSSRQ).

	**Proactiveness (PA)**	**Self-Management (SM)**	**Goal Setting (GS)**	**Mindfulness (MF)**	**Goal Attainment (GA)**	**Adjustment (AD)**	**Motivation (MO)**
(PA1)	0.838						
(PA2)	0.833						
(PA3)	0.800						
(PA4)	0.735						
(PA5)	0.714						
(PA6)	0.701						
(PA7)	0.664						
(PA8)	0.660						
(PA9)	0.628						
(PA10)	0.515						
(SM1)		0.816					
(SM2)		0.711					
(SM3)		0.697					
(SM4)		0.637					
(GS1)			0.777				
(GS2)			0.743				
(GS3)			0.678				
(GS4)			0.613				
(GS5)			0.538				
(GS6)			0.535				
(MF1)				0.793			
(MF2)				0.727			
(MF3)				0.675			
(MF4)				0.667			
(MF5)				0.618			
(MF6)				0.597			
(MF7)				0.551			
(GA1)					0.714		
(GA2)					0.656		
(GA3)					0.584		
(AD1)						0.650	
(AD2)						0.597	
(AD3)						0.571	
(AD4)						0.563	
(AD5)						0.547	
(MO1)							0.705
(MO2)							0.568
(MO3)							0.527
(MO4)							0.512

##### Factor 1—Proactiveness (PA)

There are 10 items in this factor to reflect a person’s active attitudes and behaviors to complete a task. In other words, students with the proactiveness property tend to be active in taking actions. A sample item is “(PA1) I have the courage to move out of my comfort zone and am willing to accept new challenges.” All the 10 items are positively oriented.

##### Factor 2—Self-Management (SM)

This factor contains four items examining college students’ capacity to manage themselves. Students with self-management capacities tend to well manage their time and plan to achieve goals. A sample item is, “(SM1) I do not procrastinate and can complete work by the deadline.” These four items are positively oriented.

##### Factor 3—Goal Setting (GS)

This factor includes six items that assess Taiwanese college students’ considerations when they set goals. For example, a college student may evaluate the environment and possible resources when setting up goals. A sample item is, “(GS5) I consider if this goal could help me improve myself when setting up goals”; these six items are positively oriented.

##### Factor 4—Mindfulness (MF)

A total of seven items are included in this factor to assess college students’ mindful awareness (be attentive to their actions and decisions) and their will to stick to/follow through their goals. Sample questions are: “(MF1) I give up quickly.” “(R) and (MF4) I don’t notice the effects of my actions until it’s too late.” All of the seven items are negatively oriented.

##### Factor 5—Goal Attainment (GA)

This factor contains three items that show how Taiwanese college students monitor their progress to achieve goals; in other words, they pay attention to how they are doing when they were making changes. A sample questions is, “(GA2) I set goals for myself and keep track of my progress.” All the items are positively oriented.

##### Factor 6—Adjustment (AD)

Within this factor, five items are included to describe the actions or reactions of college students when they make mistakes or encounter challenges such as admitting and learning from mistakes. Students with the adjustment trait tend to change themselves quickly to adapt to the environment. A sample question is, “(AD4) When there is a problem, I will handle and resolve it quickly.” These five items are positively oriented.

##### Factor 7—Motivation (MO)

This factor includes four items to represent college students’ motivational strategies, such as self-encouragement to help achieve goals. Students with this capacity tend to leverage their positive mindsets to confront difficulties and adversities when they complete their tasks. A sample question is, “(MO2) I set up my own reward system to increase the motivation to complete tasks.” The four items are positively oriented.

#### Confirmatory Factor Analysis (CFA)

**FIGURE 1:**
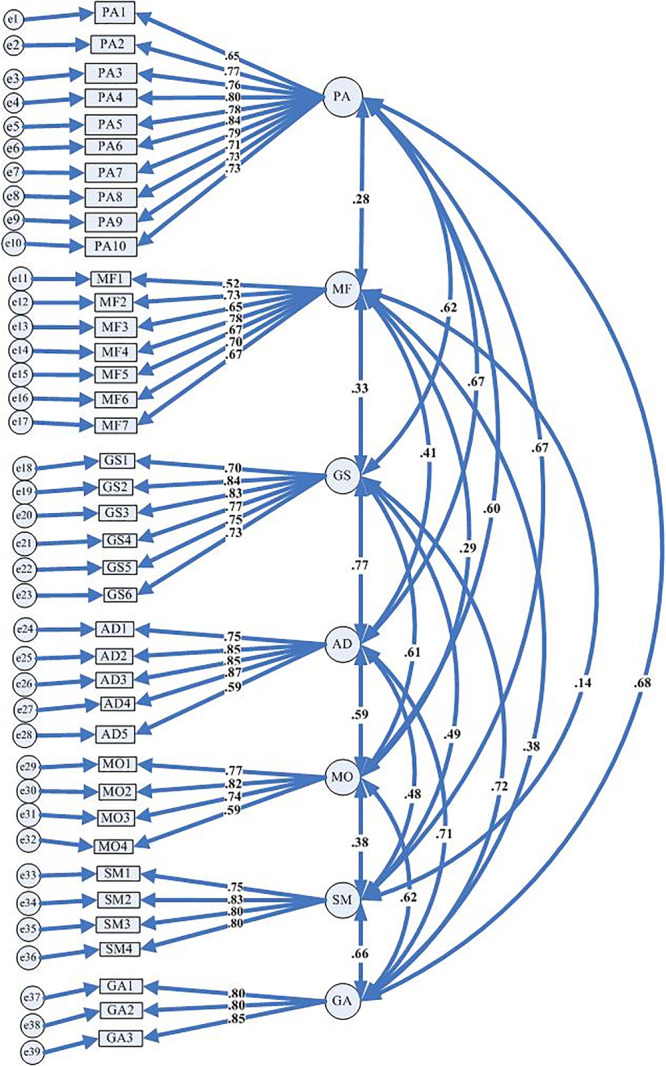
Path model and factor loadings of CFA.

We conducted a CFA with the remaining 2/3 samples (***N*** = 646) to verify the EFA model, which contained 39 items in seven dimensions. All these factors included 3 to 10 items whose factor loadings ranged from 0.52 to 0.85. Model fit results presented that Chi-square (χ^2^ = 2041.87, ***df*** = 681, ***p*** < 0.01) was significant. The values of CFI (**0**.91), RMSEA (**0**.056), TLI (**0**.906), and SRMR (**0**.047) all fell within the proper range. The entire model showed acceptable fit without any further model modification. Figure 1 presents the path model and factor loadings within each latent factor.

Aside from model fit, during CFA we also calculated the *Composite Reliability* (CR) and *Average of Variance Extracted* (AVE) for each dimension. As shown in Table 3, CR values ranged from 0.82 to 0.93, indicating good internal consistencies of the indicators. On the other hand, the AVE values lay between 0.46 and 0.67, which fell within the acceptable range of convergent validity suggested by Fornell and Larcher (1981). In addition, the square roots of AVE values (those in the diagonal with brackets) were greater than the correlations between dimensions, which indicates good divergent validity among the seven dimensions.

**TABLE 3:** Composite reliability (CR) and average of variance extracted (AVE) values of new TSSRQ.

	**PA**	**MF**	**GS**	**AD**	**MO**	**SM**	**GA**
PA	(0.758)						
MF	0.247**	(0.678)					
GS	0.588**	0.292**	(0.772)				
AD	0.627**	0.357**	0.700**	(0.789)			
MO	0.498**	0.211**	0.492**	0.473**	(0.735)		
SM	0.599**	0.137**	0.459**	0.446**	0.344**	(0.796)	
GA	0.628**	0.321**	0.674**	0.655**	0.515**	0.583**	(0.817)
CR	0.931	0.855	0.898	0.890	0.823	0.873	0.857
AVE	0.574	0.460	0.596	0.623	0.540	0.633	0.668

PA, Proactiveness; MF, Mindfulness; GS, Goal Setting; AD, Adjustment; MO, Motivation; SM, Self-Management; GA, Goal Attainment. **Coefficient is significant at the 0.01 level (2-tailed).

#### Reliability Test

Lastly, we assessed the internal consistencies of the dimensions and the entire scale. The Cronbach’s alphas were 0.93 for PA, 0.88 for SM, 0.89 for GS, 0.86 for MF, 0.85 for GA, 0.89 for AD, 0.82 for MO, and 0.95 for the total scale. The above results indicated satisfactory internal consistencies for this New TSSRQ.

### Correlations Between New TSSRQ and SPWB

In the third stage of study, we evaluated the association between New TSSRQ and SPWB. As shown in Table 4, the correlation between New TSSRQ and SPWB total scores was 0.759 (*p* < 0.001), indicating a very high association between the two constructs. Looking closer at the dimension level, EM (*r* = 0.670, *p* < 0.001), PG (*r* = 0.641, *p* < 0.001), and PL (*r* = 0.638, *p* < 0.001) in SPWB had highest correlations with New TSSRQ, which means that college students who have high capacities of SR are more likely to manage their lives, experience personal growth, and embrace clear purpose in life. On the other hand, MF, PA had highest correlations with SPWB, meaning that those with better perseverance and willpower, being more proactive in expanding horizons and experiences and skillful in managing time and preset plans are more likely to achieve better PWB. It is also notable that the correlation (*r* = 0.612, *p* < 0.001) between PA and PG, and the connections between MF and EM (*r* = 0.622, *p* < 0.001), PL (*r* = 0.615, *p* < 0.001) achieved the highest values: both were higher than 0.60.

**TABLE 4:** Correlations between new TSSRQ and Scale of Psychological Well-being (SPWB).

	**PA**	**SM**	**GS**	**MF**	**GA**	**AD**	**MO**	**TSSRQ**
AT	0.531***	0.402***	0.338***	0.434***	0.249***	0.374***	0.254***	0.547***
EM	0.487***	0.533***	0.494***	0.622***	0.417***	0.407***	0.352***	0.670***
PG	0.612***	0.348***	0.450***	0.527***	0.391***	0.401***	0.344***	0.641***
PR	0.394***	0.230***	0.384***	0.370***	0.299***	0.354***	0.351***	0.477***
PL	0.499***	0.404***	0.466***	0.615***	0.404***	0.388***	0.329***	0.638***
SA	0.530***	0.444***	0.354***	0.462***	0.293***	0.353***	0.392***	0.586***
SPWB	0.650***	0.504***	0.529***	0.647***	0.437***	0.485***	0.432***	0.759***

***Coefficient is significant at the less than 0.01 level (2-tailed).

Regarding gender differences, our findings from independent sample *t*-tests indicated that females outperformed males in MF (*t* = −2.14, *p* < 0.05) and MO (*t* = −2.33, *p* < 0.05) dimensions of New TSSRQ. For SPWB, males obtained significantly higher scores in AT (*t* = 2.14, *p* < 0.05) than females. On the contrary, females scored higher than males in terms of EM (*t* = −3.45, *p* < 0.05), PG (*t* = −5.55, *p* < 0.05), and PL (*t* = −4.37, *p* < 0.05) dimensions.

## Discussion

### Discussion of New TSSRQ

In this study, we identified seven factors of SR, of which all the original five factors were retained, namely GA, MF, AD, PA, and GS. While the dimensions are identical, many item narrations in the new version are more granulated and specific. For instance, an item in the original GS factor is, “I have a hard time setting goals for myself,” whereas in the new version, a GS item is presented as, “GS1- I evaluate the environment and possible resources I can receive when setting up goals.” In addition, items in the PA and GS factors are much expanded, which better captures various representations of a proactive person, as well as what a person may think or do when setting goals. On the other hand, the number of items in Goal Attainment had reduced from 7 to 3. When we compared items in both versions, we found that the new PA items became more focused on/refined to tracking progress and following up a pre-set plan, and those less relevant items, such as “I have personal standards and try to live up to them” and “Once I have a goal, I can usually plan how to reach it” are no longer included in the GA dimension.

In addition to the original five dimensions, two more factors were obtained in the New TSSRQ; one is the motivation strategy (MO) based on Pintrich’s (2004) theorizing as mentioned earlier. The other, the self-management (SM) dimension, delineates how a person manages time, follows through a plan without getting distracted, and possesses a good self-control capability. The SM dimension is well aligned with the “potential for control” assumption of SR (Pintrich’s, 2004), making the new scale more theoretically solid. Furthermore, the GA dimension resembles the “monitoring” stage of SR and this newly created SM resembles the “controlling” stage. Together, the two stages are termed as “volitional control” (Pintrich’s, 2000). While Pintrich et al. (2000) argued that the monitoring and controlling are usually hard to separate in some assessment instruments (we did yield a monitoring/controlling dimension in a previous study: Chen and Jang, 2019), here we obtained separate dimensions of monitoring and controlling in this new TSSRQ. To some extent, this means that our New-TSSRQ may embrace a better construct validity in terms of Pintrich’s (2000, 2004) four-stage theorizing.

In summary, in this study, we enriched items in GA, GS, and AD dimensions, further include motivation aspect of SR, and incorporate student perspectives of SR via open-ended questions. As shown in the results section, EFA, CFA, and subsequent reliability tests showed adequate results, and the separation of SM and GA dimensions aligns better with the monitoring and controlling phases of Pintrich’s (2000). Together, we deem the revised New TSSRQ to be a theoretically solid, statistically reliable, and contextually adaptive instrument to measure Taiwanese college students’ SR.

### Correlations Between New TSSRQ and PWB

In this study, we explored the correlation between New TSSRQ and PWB and yielded a very high correlation (*r* = 0.769, *p* < 0.001). This result is consistent with prior studies such as Mattern and Bauer (2014), Durand-Bush et al. (2015), and Singh and Sharma (2018). Together, these studies enlighten the path for promoting PWB through the cultivation of SR. To leverage SR in educational settings, Boekaerts and Corno (2005) organized three categories of intervention, including (1) cognitive-behavior modification (e.g., goal setting and feedback), (2) direct instruction in metacognitive skills and strategies, and (3) strategies based on principles of socioculturalism (e.g., collaborative learning). Similarly, Zumbrunn et al. (2011) proposed four directions to promote SR in the classroom, including (1) direct instruction and modeling, (2) guided and independent practice, (3) social support and feedback, and (4) reflective practice. Following these guidelines, university teachers can carefully consider student characteristics, content properties, and specifications in the context, and tacitly integrate various SR strategies, such as team-based collaborative learning (Panadero et al., 2015), peer coaching (Asghar, 2010), reciprocal feedback (Schünemann et al., 2017), and diaries/reflection journals (Winne, 2005) to promote college students’ self- and co-regulation.

More specifically, the PA and MF dimensions correlated highest with Taiwanese college students’ PWB. A follow-up stepwise regression analysis also indicated that MF was the strongest predictor of PWB, followed by PA. As such, more emphasis could be laid on strengthening college students’ mindfulness and proactiveness in their course of study. In the literature, a number of mindfulness-based intervention (MBI) strategies, such as body scan, breathing, and thought-watching practices, have been carried out in school curricula to promote students’ attention and affective/behavioral SR (Semple et al., 2017). Such mindfulness approaches, according to the Garrison Institute report (Jha, 2005), are comparatively easy to learn, and they may help students become more focused and experience fewer distractions. While at present most MBI strategies were carried out in k-12 classrooms, more studies are recommended to test their applicability in college settings.

Besides concentration and mindful awareness, in this study mindfulness also includes resolution, persistence, and perseverance (see items MF1, MF2, MF3, and MF6) which is close to the concept of *Grit*, defined as “passion and perseverance for long-term and meaningful goals” (Duckworth et al., 2007; Duckworth, 2016). In her popular book, *Grit: The power of passion and perseverance*, Duckworth (2016) proposed four ways to promote Grit, including (1) develop *interests* that induce inner passion; (2) *practice* to keep improving until mastery; (3) ascertain *purposes* and embrace high goals to benefit the self and others; and (4) lean to *hope* when facing difficulty and adversity. Above all, the “Growth Mindset” is crucial to set out the change process, generate impetus to move on, and become more adaptive and resilient when facing difficulties. In addition to the above inside-out approach, significant others, such as parents, teachers, and peers are important to provide support, respect, and optimal demand that help individuals to internalize norms, values, and high standards.

As with promoting college students proactiveness, Deci and Ryan’s (1985), Deci and Ryan’s (2002) self-determination theory is insightful. According to SDT, *autonomous* individuals not only embrace intrinsic motivation to act, but they also identify or integrate extrinsic goals and values to the self, especially when their tasks are not inherently interesting. Following SDT, Reeve and Jang (2006) proposed 11 autonomy-supportive instructional behaviors of teachers, such as asking what students want, providing rationales of the task, praise as informational feedback, and offering encouragement to achieve goals. Such strategies, ideally coupled with abovementioned SR strategies and authentic learning tasks (e.g., problem-based learning), would help students assume more responsibilities of their duties, become more aware of their true needs and aspirations during goal setting and attainment, reflect on the meanings and values of the task, and strengthen their self-efficacy to achieve their goals. In addition to inner motivations, Parker et al. (2010) pointed out exterior factors that affect individuals’ proactiveness, such as job enrichment, job control, leadership, and interpersonal climate. It is suggested that college instructors consider these factors and conditions, and design a learning environment that is interesting, inspiring, and encouraging.

Beyond formal learning, college students are encouraged to broaden their horizon and perspectives by participating in a variety of experiential learning activities. In Taiwan, since 2009, the Youth Development Administration, Ministry of Education had launched the “Youth Travel in Taiwan” program, in which many “Youth Travel Spots” across Taiwan were established to provide learning opportunities categorized in culture, tribe, ecology, rural and fishing villages, volunteer services, and physical fitness^1^. Furthermore, each year the government subsidizes youth teams to carry out their travel learning plans that are profound, creative, and contributive to the society. Such initiatives help college students step out of their comfort zones to embrace new challenges, explore and cultivate multiple interests, and work independently and collaboratively to resolve problems during experiential learning. Relating to items PA1, PA2, PA3, PA4, and PA6, such experiential learning has great potential to promote college students’ proactiveness in their study, life, and beyond.

## Conclusion

In this study, we expanded items and enriched perspectives of the original TSSRQ, particularly student perspectives, and affective aspects of SR were covered in this revision. The 7-factor, 39-item instrument would serve as an ideal tool to assess Taiwanese college students’ cognition, motivation, and behaviors of SR. Other than assessment purposes, the New TSSRQ can be used as a self-reflection tool for college students to ruminate on their status of SR, thereby designing their own schedule for self-growth and promotion.

In addition to scale validation, another contribution of this study is to verify the correlation between SR and college students’ PWB. Our finding of salient correlations between scale dimensions would shed light on ways to promote college students’ proactiveness, mindfulness, and SR in general. Moreover, females obtained significantly higher SR scores in MF and MO, and greater SPWB scores in EM, PG, and PL than males. On the contrary, males significantly outperformed females in AT of SPWB. It is advised that educators identify and verify effective strategies to support college students’ SR through intra- and extracurricular activities, with the higher goal to promote college students’ positive developments and well-being. Future studies can also examine gender differences in SR and PWB in more detail (perhaps through qualitative approaches) to identify factors or mechanisms that differentiate males and females in MF, MO, EM, PG, PL, and AT dimensions.

This study has limitations; foremost is the self-report nature of New TSSRQ and SPWB that are susceptible to social desirability bias (Fisher, 1993). Future studies may also incorporate qualitative approaches such as observation or interviews, as suggested by Dinsmore (2017), to triangulate findings. In addition, while we have strived to enrich items in this new revision, the GA (Goal Attainment) dimension only contains three items, which to some extent limit the representativeness of the factor. Future studies may further add items in this dimension and reconfirm the psychometric quality of the New TSSRQ. It is also recommended that future studies investigate the applicability of the scale to other groups and regions, such as secondary students and adults, and those in other Asian countries with similar cultural backgrounds. Last, but not least, we hope this new measurement of general trait self-regulation could inspire self-regulation research in extended topics and contexts and continue to explore ways to leverage individuals’ positive and healthy functioning, life adjustment, and PWB throughout their life span.

## Data Availability Statement

The datasets generated for this study are available on request to the corresponding author.

## Ethics Statement

Ethical review and approval was not required for the study on human participants in accordance with the local legislation and institutional requirements. Written informed consent for participation was not required for this study in accordance with the national legislation and the institutional requirements.

## Author Contributions

Y-HC designed the entire research study, collected and analyzed the data, and completed the manuscript. Y-JL assisted in study design and data analysis, and to complete the manuscript. Both authors contributed to the article and approved the submitted version.

## Conflict of Interest

The authors declare that the research was conducted in the absence of any commercial or financial relationships that could be construed as a potential conflict of interest.

## Footnotes

1 https://youthtravel.tw/en/travelspot_location.php

## Appendix: New Taiwanese Short Self-Regulation Questionnaire (NTSSRQ)

### Proactiveness (PA)

PA1- I have the courage to move out of my comfort zone and am willing to accept new challenges.

PA2- I take chances and even create opportunities for myself.

PA3- I actively develop a variety of interests.

PA4- I would actively expand my network and various resources.

PA5- I have courage to express my opinions.

PA6- I have courage to put my thoughts into action.

PA7- I actively ask experienced people questions in order to increase my knowledge and ability.

PA8- I actively learn in order to enhance myself.

PA9- When I think to change one thing, I usually can discover different possibilities.

PA10- I have my own opinions and thoughts on everything.

### Self-Management (SM)

SM1- I do not procrastinate and can complete work by the deadline.

SM2- I can arrange and manage my time.

SM3- I follow up my plans to do things without being easily influenced by other people or factors.

SM4- I am able to self-manage and have good self-control abilities.

### Goal Setting (GS)

GS1- I evaluate the environment and possible resources I can receive when setting up goals.

GS2- I consider the cost for this goal and benefits I can receive when setting up goals.

GS3- I consider my abilities and physical and mental status when setting up goals.

GS4- I estimate if this goal is feasible or not when setting up goals.

GS5- I consider if this goal could help me improve myself when setting up goals.

GS6- I look for information and acquire relevant knowledge in order to achieve goals.

### Mindfulness (MF)

MF1- I give up quickly. (R)

MF2- I have trouble following through with things once I’ve made up my mind to do something. (R)

MF3- I put off making decisions. (R)

MF4- I don’t notice the effects of my actions until it’s too late. (R)

MF5- I get easily distracted from my plans. (R)

MF6- I have trouble making up my mind about things. (R)

MF7- Most of the time I don’t pay attention to what I’m doing. (R)

### Goal Attainment (GA)

GA1- I usually keep track of my progress toward my goals.

GA2- I set goals for myself and keep track of my progress.

GA3- When I’m trying to change something, I pay attention to how I’m doing.

### Adjustment (AD)

AD1- I am willing to admit and reflect my mistakes once I find or make errors.

AD2- When I discover mistakes, I reflect to avoid making the same mistake again.

AD3- I learn from mistakes.

AD4- When there is a problem, I will handle and resolve it quickly.

AD5- When I encounter any problems, I would look for reasons causing them.

### Motivation (MO)

MO1- When I am uncomfortable or in a lack of motivation, I say something inspiring to encourage myself.

MO2- I set up my own reward system to increase the motivation to complete tasks.

MO3- I imagine what success would be like to encourage myself when I work on one task.

MO4- When I work on tasks, I provide self-affirmation to myself in terms of small achievements in the process in order to maintain ongoing confidence and motivation.
